# Desertification Sensitivity Analysis Using MEDALUS Model and GIS: A Case Study of the Oases of Middle Draa Valley, Morocco

**DOI:** 10.3390/s18072230

**Published:** 2018-07-11

**Authors:** Atman Ait Lamqadem, Biswajeet Pradhan, Hafid Saber, Abdelmejid Rahimi

**Affiliations:** 1 Laboratory of Geodynamic and Geomatic, Department of Geology, Faculty of Sciences, 24010 Chouaïb Doukkali, Morocco; aitlamqadem.a@ucd.ac.ma (A.A.L.); Hafidsaber@yahoo.fr (H.S.); mjidrahimi@yahoo.fr (A.R.); 2 Centre for Advanced Modelling and Geospatial Information Systems (CAMGIS), Faculty of Engineering and Information Technology, University of Technology Sydney, Sydney, NSW 2007, Australia; 3 Department of Energy and Mineral Resources Engineering, Choongmu-gwan, Sejong University, 209, Neungdong-ro Gwangin-gu, Seoul 05006, Korea

**Keywords:** MEDALUS, GIS, Sentinel-2 data, remote sensing, Middle Draa Valley, Morocco

## Abstract

Oases can play a significant role in the sustainable economic development of arid and Saharan regions. The aim of this study was to map the desertification-sensitive areas in the Middle Draa Valley (MDV), which is in the southeast of Morocco. A total of 13 indices that affect desertification processes were identified and analyzed using a geographic information system. The Mediterranean desertification and land use approach; which has been widely used in the Mediterranean regions due to its simplicity; flexibility and rapid implementation strategy; was applied. All the indices were grouped into four main quality indices; i.e., soil quality; climate quality; vegetation quality and management quality indices. Each quality index was constructed by the combination of several sub-indicators. In turn; the geometric mean of the four quality index maps was used to construct a map of desertification-sensitive areas; which were classified into four classes (i.e., low; moderate; high and very high sensitivity). Results indicated that only 16.63% of the sites in the study were classified as least sensitive to desertification; and 50.34% were classified as highly and very highly sensitive areas. Findings also showed that climate and human pressure factors are the most important indicators affecting desertification sensitivity in the MDV. The framework used in this research provides suitable results and can be easily implemented in similar oasis arid areas.

## 1. Introduction

Drylands (arid, semi-arid, and dry sub-humid areas) cover approximately 40% of the Earth’s surface [1]. Desertification refers to land degradation caused by climate change and human activity in arid, semi-arid, and dry sub-humid areas [2]. Africa is a degraded continent. More than 46% of Africa’s surface is affected by desertification. In Morocco, a large part of the land (approximately 90%) is classified as desert [3]. Human pressures, dry climate, and natural hazards (such as landslides and soil erosion) are the main driving factors of desertification in Morocco.

Desertification is a complex phenomenon resulting from the interaction of natural (biophysical) and anthropogenic factors with different temporal and spatial variabilities [4]. Describing and assessing the state of current desertification requires key variables and indicators that consider biophysical and human factors.

Field-based studies are one of the assessment methods of land degradation and desertification. With the advent development of geospatial tools and remote sensing techniques, field-based studies have been popularly used as a powerful tool for the monitoring and assessment of arid lands and their dynamics. Compared with field-based studies, remote sensing-based assessment is cost effective and time efficient for land degradation risk mapping [5,6]. 

In the last decades, several approaches and methods have been developed to identify areas of land degradation. Some of them use spectral biophysical indicators [7,8,9] and other integrating anthropogenic indicators [10]. To represent the desertification process in a comprehensive and easy way, a conjugal methodology can be used by taking into consideration quantitative and qualitative methods and by using specific indicators [11,12].

The Mediterranean desertification and land use (MEDALUS) approach identifies environmentally sensitive areas (ESAs) through the Environmentally Sensitive Area Index (ESAI) [10]. The ESAI is implemented by considering several variables, i.e., physical (soil quality), environmental (vegetation quality), climatic (climate quality), and social (management quality) indicators. This index can be used to obtain an in-depth understanding of the parameters causing the desertification threat at a certain point. This approach is simple, robust, widely applicable, and acceptable to new indicators and parameters and can be adjusted to several level scales [13].

The MEDALUS model has been a widely recognized approach in different Mediterranean regions at national, regional, and local scales. It was used in an entire Greek state to assess desertification sensibility using the four indicators recommended by the original MEDALUS report [14]. Ladisa et al. (2012) assessed desertification sensibility in the Apulia region (southeastern Italy) using this method, and the results indicated good performance for this technique [4]. In another work, Trotta et al. (2015) applied MEDALUS at a local scale in Castel Porziano (central Italy) [15]. Similarly, Contador et al. (2009) applied this method in Extremadura (southwestern Spain) [16]. In a separate paper, Symenoakis assessed sensitivity to land degradation and desertification using Environmental Sensitive Area Index at Lesvos Island [17]. In Lebanon, the method was applied in an arid region by adding certain parameters (i.e., rock hardness, permeability, soil organic matter, clogging, and erodibility) and excluding others (i.e., texture parent material and soil depth) [18]. The method has also been applied in Mediterranean African countries, such as Algeria [19]. In Morocco, the approach was applied in the arid regions of the Sous Massa River Basin to propose an action plan of potential interventions to mitigate the desertification problems in this region [20] and in Oued El Maleh, central Morocco [21]. However, the MEDALUS model has been elaborated and developed in the context of Mediterranean areas prone to desertification, and most applications have been done in semi-arid, arid, and hyper-arid zones. The model was adopted in the same climate context of the study area, which can be considered to be a hyper-arid climate. For example, Benmessaud assessed a desertification sensitive area in the Biskra region (South Aurès) in Algeria using the MEDALUS model [22]. Similarly, Benabderrahmane also used the same approach for mapping the desertification sensitive at the Eastern Algeria (Aures region) [23].

This study aims to map the desertification-sensitive areas in the MDV on the basis of the four following indicators: soil, climate, vegetation, and human pressure. Each indicator was constructed by considering various sub-indicators (i.e., parameters). This approach is inspired by the original MEDALUS approach [10].

### 2. Materials and Methods

#### 2.1. Study Area

**Figure 1:**
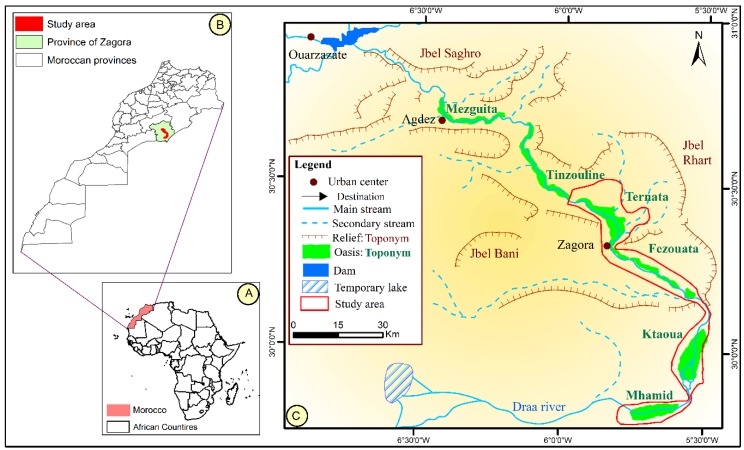
(**A**) Location of Morocco in Africa. (**B**,**C**) Location of the study area in Zagora Province.

Administratively, the MDV is a part of Zagora Province, which includes two urban and 18 rural communities. Geographically, the MDV is located in the southeast of Morocco and in the south of the High Atlas mountains [24]. The bed of Draa River forms a chain of six successive oases from upstream to downstream: Mezguita, Tinzouline, Ternata, Fezouata, Ktaoua, and Mhamid (Figure 1). The altitudes of the areas vary between 500 and 1000 m. It is located in the middle of the 6° west meridian and below the 30° north parallel. The basin of Draa has an area of approximately 14,380 km^2^ and a width of 1200 km, crossed by Wadi Draa (ephemeral river) and fed by the El Mansour Eddahbi Dam [24].

The Saharan region has an arid climate, with an average value of the aridity index of 0.03 [25]. The average rainfall varies from 54 mm to 108 mm, and the evaporation reaches 3000 mm/year. The maximum temperature can reach more than 48 °C during summer, and the minimum temperature varies between −1 °C and 7 °C during winter. 

The vegetation coverage of oases is characterized by three main types: palm trees, fruit trees and seasonal agriculture. Due to the arid climate, the hydrological system depends, to a certain extent, on the water runoff in the High Atlas mountain chain, El Mansour Eddahbi Dam and generally from wells. 

The Middle Draa Valley (MDV) is an arid area located in south-eastern Morocco. This area has an arid climate (high temperature and low rainfall), wind erosion and water shortage. In recent years, the MDV has encountered severe environmental pressure from agricultural practices that are inadequate for the arid climate, and several environmental problems, such as wind and water erosion. Despite these degradations, only a few studies have been conducted in this area. Geologically, the MDV is a part of the domain of Palaeozoic formation and Bani (the Ordovician Mountain), whose geological formations belong to varied ages from the Precambrian to the Quaternary [26].

In this research, the last four oases (Ternata to Mhamid) were chosen as the study areas (Figure 1). These oases are classified as the most degraded in Morocco [27].

#### 2.2. Data Used

To map the desertification-sensitive areas in the MDV, a list of data was used and processed as follows.

- The ASTER digital elevation model (DEM) was used to retrieve the slopes and aspect gradients. The data (spatial resolution of 30 m) are available at https://lpdaac.usgs.gov/.
- Demographic data of the National Census of the Population and Habitat of 2014 were acquired from the Moroccan High Commission for Planning. The data are available at https://www.hcp.ma.
- The census of livestock was collected from the Regional Centre of Agricultural Development of Ouarzazate (ORMVAO).
- Historical data of precipitation (1980–2015) were also collected from ORMVAO, which included a time series of the monthly precipitations of Ternata and Ktaoua climatological stations.
- Raster data of precipitation with 1 km^2^ were also used in this research. This data were an average of the monthly precipitations from 1970 to 2000 [28]. The data are available at http://www.worldClim.org.
- Data related to aridity were collected from the Consortium for Spatial Information (CSI), who provides high-resolution global raster climate data with a 1 km^2^ spatial resolution. The data are available at http://www.cgiar-csi.org.
- Soil depth data were extracted and reproduced from the “integrated approach to the efficient management of scarce water resources in West Africa” (IMPETUS) project via http://www.impetus.uni-koeln.de.
- A geological map of Hamada Draa, with a scale of 1:200,000, was acquired from the Moroccan Minister of Energies, Mines and Sustainable Development and was used to extract the parental material (lithological formations).
- In this study, a Sentinel-2 (S-2) space-borne satellite image was used to extract vegetation coverage and land use/cover map of the study area. The image was acquired on 3 July 2017. S-2 imagery was captured using a multispectral imaging sensor that uses the push-broom imaging technique to measure the Earth’s top-of-atmosphere reflected radiance. Thirteen bands (443–2190 nm) were present [29]. The level 1C 12-bit encoded S-2 image was freely downloaded from the Copernicus Open Access Hub at https://scihub.copernicus.eu/. The pre-processing of this data included radiometric and geometric correction and orthorectification (ortho-images in UTM/WGS84 projection) using Planet 90 m resolution DEM [30]. The free SNAP tool developed by the European Space Agency was used to convert the level 1C image to level 2A bottom-of-atmosphere reflected values [31], with the association of the Sen2Cor tool [32] for the atmospheric correction. The pre-processing also included resampling of bands to 10 m of spatial resolution using the nearest neighbour algorithm and then clipping of the site of study from the scene.
- Ground truth data were collected from the MDV to validate the land use/cover classification using the S-2 image and the final map of the desertification-sensitive areas. The water samples were also collected from wells in different oases to calculate water salinity.

#### 2.3. Methodology

Desertification is a complex phenomenon that leads to the reduction of land productivity and interaction of time and space [33]. This phenomenon is closely linked to several environmental factors (climate, soil, vegetation and morphology) and anthropogenic activities (human behaviour and socio-economic activities). 

The key indicators of the MEDALUS model identify ESAI as an output, which defines the ESAs. Generally, ESAs represent areas whose socio-economic and ecological aspects are not sustainable for a particular environment [34]. The evaluation of ESAs is based on a combination of the physical and anthropogenic indicators. The physical properties describe the environmental conditions of lands, including soil, climate, and vegetation qualities. The management quality index (MQI) is calculated by considering the human and animal pressures to the environment related to the desertification process. A geodatabase and several thematic maps were prepared and standardized with the same projection system and spatial resolution.

**Figure 2:**
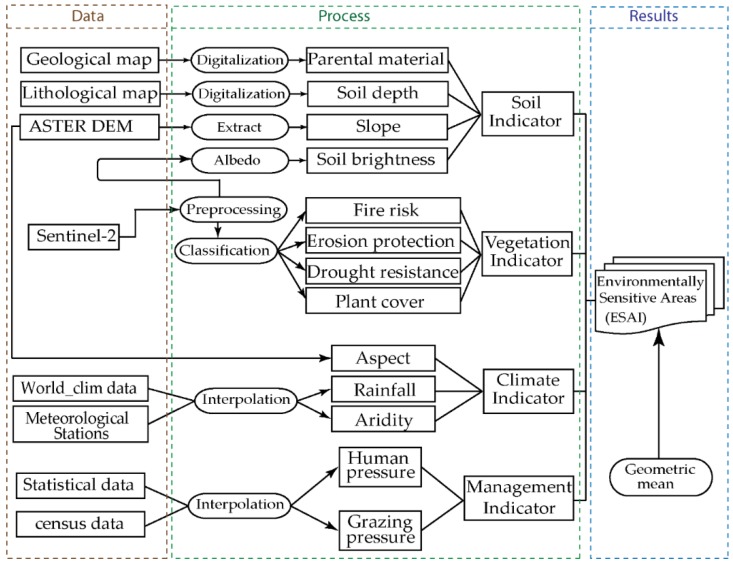
Methodological flowchart.

The indicators were grouped into four quality indices, namely, the soil quality index (SQI), climate quality index (CQI), vegetation quality index (VQI), and MQI (Figure 2). Each indicator was calculated by means of sub-indicators, which were also classified. For each class, a weight value was allocated. The values of the weights varied from 1 (least sensitive to desertification), to 2 (most sensitive to desertification), and values between 1 and 2 represented relative vulnerability [10]. The final ESAI map was produced by geometric mean in a geographical information system (GIS). All raster layers of this project were resampled to 30 m of the spatial resolution using the nearest neighbourhood method and projected to a UTM zone 30 projection system.

##### 2.3.1. SQI

Soil is a key parameter that intervenes in the assessment of desertification. Soil quality assessment depends on lithological formation, soil depth, topographical slope, organic matter, and soil texture. In this step, maps and data related to soil are collected and reproduced. Soil depth was reproduced after the results of the IMPETUS project. The slope gradient map was retrieved from ASTER DEM. The geological map was exploited to extract different parental materials (lithological formation). Then, the parental material was classified into three classes according to the coherence of the lithology. 

In this research, soil brightness was integrated as an indicator that influences soil quality. In general, soils rich in organic matter are represented in remotely sensed images in dark colours. By contrast, bright colours correspond to non-developed soils (poor in organic matter) [31]. Surface albedo was used to extract soil brightness using Liang albedo [35]. Albedo is widely used as an indicator to assess desertification in arid and semi-arid areas [7,9,33,34,35,36,37,38,39]. Surface albedo was retrieved using S-2 imagery after pre-processing.

SQI was calculated by using the following formula (Equation (1)):

(1) $$\mathrm{SQI}={\left(S\times \mathrm{Dp}\times \mathrm{Pm}\times B\right)}^{1/4},$$

where SQI is the soil quality index, *S* is the topographical slope, DP is the horizontal depth of soil, PM is the parental material and *B* is the brightness of soil (surface albedo).

##### 2.3.2. VQI

Vegetation coverage plays an important role in the assessment of desertification in arid and semi-arid areas. Vegetation can decrease sand dune encroachment intensity [40] and soil erosion and create a micro-climate for the local population and their livestock [41,42]. Therefore, vegetation plants enrich organic soil and provide a high capacity to reduce the intensity of soil erosion, thus improving the coherence and quality of soil. The basic source needed for the elaboration of data layers related to vegetation indicator is the land use/cover map of the MDV. This map was derived from S-2 imagery by using supervized classification with maximum likelihood classification algorithm [43]. The resultant map was validated using field data with matrix confusion and Kappa coefficient [44]. The classified map was resampled from 10 m to 30 m using nearest neighbour resampling to obtain the same spatial resolution of all raster layers of indicators [45]. The land use/cover map was used to elaborate fire risk, drought resistance and erosion protection maps, which constitute the VQI. Subsequently, normalized difference vegetation index (NDVI) [46] was used to elaborate the plant cover map. The VQI was calculated using Equation (2).

(2) $$\mathrm{VQI}={\left(\mathrm{Fr}\times \mathrm{Ep}\times \mathrm{Dr}\times \mathrm{Pc}\right)}^{1/4},$$

where Fr is the fire risk, Ep is the erosion protection, Dr is the drought resistance and Pc is the plant cover.

##### 2.3.3. CQI

Climate variability, succession of drought periods and severe climate conditions (low precipitations and high temperatures) can make plants and lands vulnerable to desertification. Water availability depends on the value of the average annual precipitation. In the zones where the precipitation is less than 280 mm/year, soil and wind erosion and degradation of lands are severe [10]. In the study area, the annual average precipitation is generally lower than 100 mm/year. The aridity, which is generally related to rainfall and evapotranspiration and/or temperature, is a crucial environmental factor. The combination of high temperatures and low rainfall affects water availability and consequently influences vegetation growth and soil moisture [47]. 

One of the most important parameters affecting the micro-climate quality is slope aspect, and soil moisture is related to the aspect direction. An aspect with southeast orientation receives more sunlight than does a northeast one. Furthermore, high sunlight implicates low surface moisture, high evaporation and vegetation degradation and, implicitly, water, and wind erosion [10,13]. However, low humidity is presented by south, southeast, and southwest exposures, whereas high humidity corresponds to north, northeast, and northwest exposures [48]. 

The CQI was evaluated by considering the following factors: rainfall, aridity extracted on the basis of climate and morphological data and aspect (Equation (3)).

(3) $$\mathrm{CQI}={\left(R\times \mathrm{AI}\times A\right)}^{1/3},$$

where is rainfall (mm), AI is the aridity index and A is the aspect direction.

The rainfall map was produced by using the data provided by WorldClim [28]. The data included the mean monthly rainfall for 1970–2000 (12 bands, each representing a month). 

The Potential Evapo-Transpiration (PET) and the aridity index layers were given and calculated by Trabucco [25], by the exposition of WorldClim temperatures and precipitations data. The Hargreaves method was applied to calculate the PET [49]. The method uses mean monthly temperature (Tmean), mean monthly temperature range (TD) and mean monthly extra-terrestrial radiation (RA, radiation on top of atmosphere) to calculate mean PET, as shown below(Equation (4)) [25,49]:

(4) PEF=0.0023×RA×(Tmean+17.8)×TD×0.5(mm/day)

The mean aridity index was calculated by using Equation (5) [47,48].

(5) Aridity Index (AI)=MAP/MAE,

where MAP is the mean annual precipitation and MAE is the mean annual potential evapotranspiration.

The aspect map was retrieved directly from ASTER DEM and then reclassified into two classes.

##### 2.3.4. MQI

Anthropogenic pressure affects ecosystem vulnerability. Deforestation and land degradation are linked to agro-sylvo-pastoralism activities, overgrazing, logging, and inadequate agricultural practices. In this study, MQI was calculated using human and grazing pressures. Human pressure was calculated using the number of inhabitants of each administrative community, whereas grazing pressure grazing was calculated using the number of livestock (Equation (6)).

(6) $$\mathrm{MQI}={\left(\mathrm{Human}\;\mathrm{pressure}\times \mathrm{Grazing}\;\mathrm{pressure}\right)}^{1/2}$$

##### 2.3.5. ESAI

The ESAI was given by the combination of the four indices described above. SQI, VQGI, CQI and MQI were classified and weighted into three classes. The maps of the four indicators were combined to calculate the ESAI using the following formula (Equation (7)):

(7) $$\mathrm{ESAI}={\left(\mathrm{SQI}\times \mathrm{CQI}\times \mathrm{VQI}\times \mathrm{MQI}\right)}^{1/4}$$

## 3. Results

In this part of the study, the evaluation of the four indexes of the ESA approach is elaborated in detail. The weight values in the tables are related to the influence on the desertification process, ranging from 1 (low sensitivity) to 2 (high sensitivity).

### 3.1. SQI

Soil is connected to water availability and erosion threat. The SQI was calculated through the combination of different sub-indicators indicated in Equation (1). A large part of the oases in the MDV are located in a quaternary formation. The quaternary part includes terraces of major bed, mobile sand, unconsolidated scree, low terraces, dejection cones, and white limestones, probably lacustrine and often conglomeratic. Furthermore, the Ordovician part contains sandstone, quartzites, and claystone. Soils derived from different parent materials react differently to erosion, absorbency and production of biomass: the presence of pebbles still causes an increase in runoff and therefore improved safeguard from desertification. 

Soil depth is linked to water availability. A deep soil can assure water reserves and can then provide a good condition for vegetation development and growth.

The erosion process is directly linked to slope gradient. Soil erosion increases with high slope gradient and rainfall and decreases with low slope gradient. 

The influence of each class for each sub-indicator constructs SQI that was weighted based the influence on desertification process (Table 1). 

**Figure 3:**
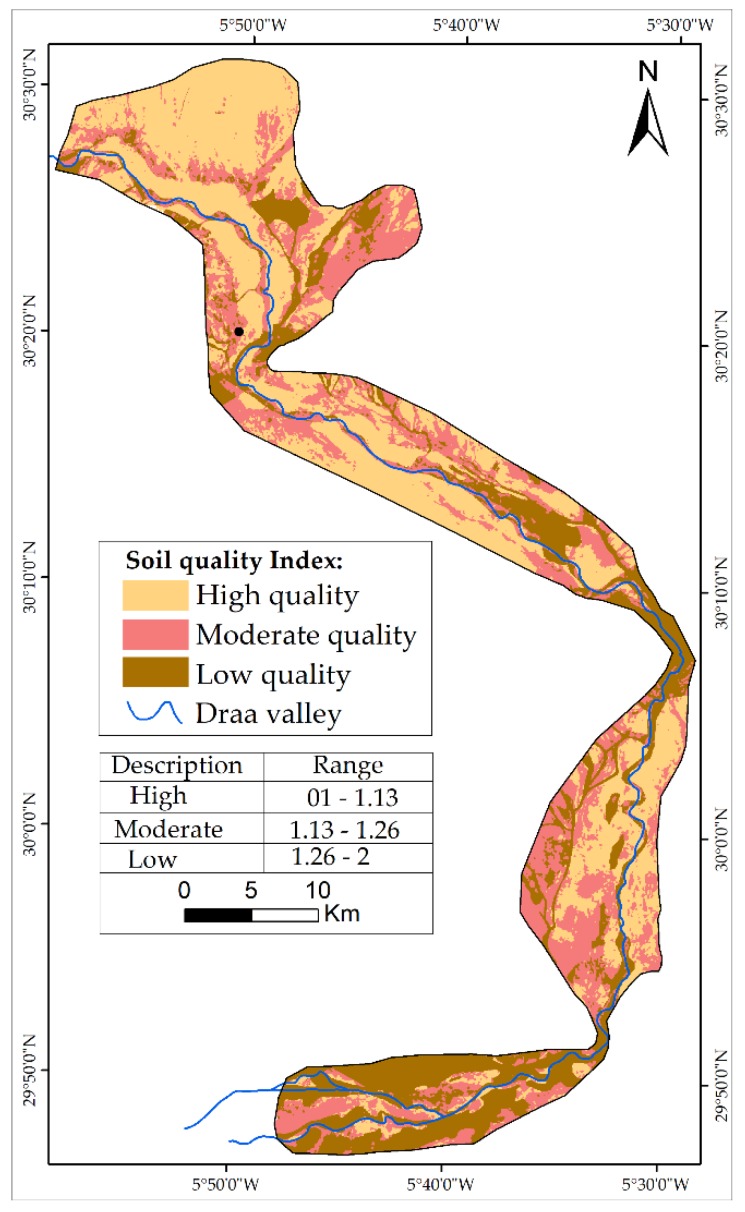
Map of SQI.

The results of the SQI indicated that approximately 43.21% of the study area is classified with high quality and 25% with low quality. The map of SQI was produced by the geometric mean of the four sub-indicators (Equation (1)), and then the map was classified into three classes (Figure 3). The results indicated that the zones with low soil quality are located in the south part of the MDV (Mhamid oasis), with a large part of this oasis consisting of sand dunes.

### 3.2. CQI

The study area contains only two climatic stations (Ktaoua and Ternata). The two stations cannot provide a good distribution of precipitation estimate for the entire study area. For this reason, data from the WorldClim database were used to calculate CQI. The rainfall is characterized by the variability in space and time and a low average rainfall. Annual average rainfall varies between 1950 and 2000 in the study area and ranges from 50 mm to 100 mm. This low rainfall leads to high aridity, and the values of the aridity index (calculated using Trabuco formula) extend from 0.023 to 0.039.

CQI was calculated by the combination of three sub-indicators, which were classified and weighted according to the values in Table 2. 

Approximately 42.76% of the study area is classified with low CQI and 27.43% with high CQI, explaining that climate indicator is one of the main factors that affects the desertification in the MDV.

**Figure 4:**
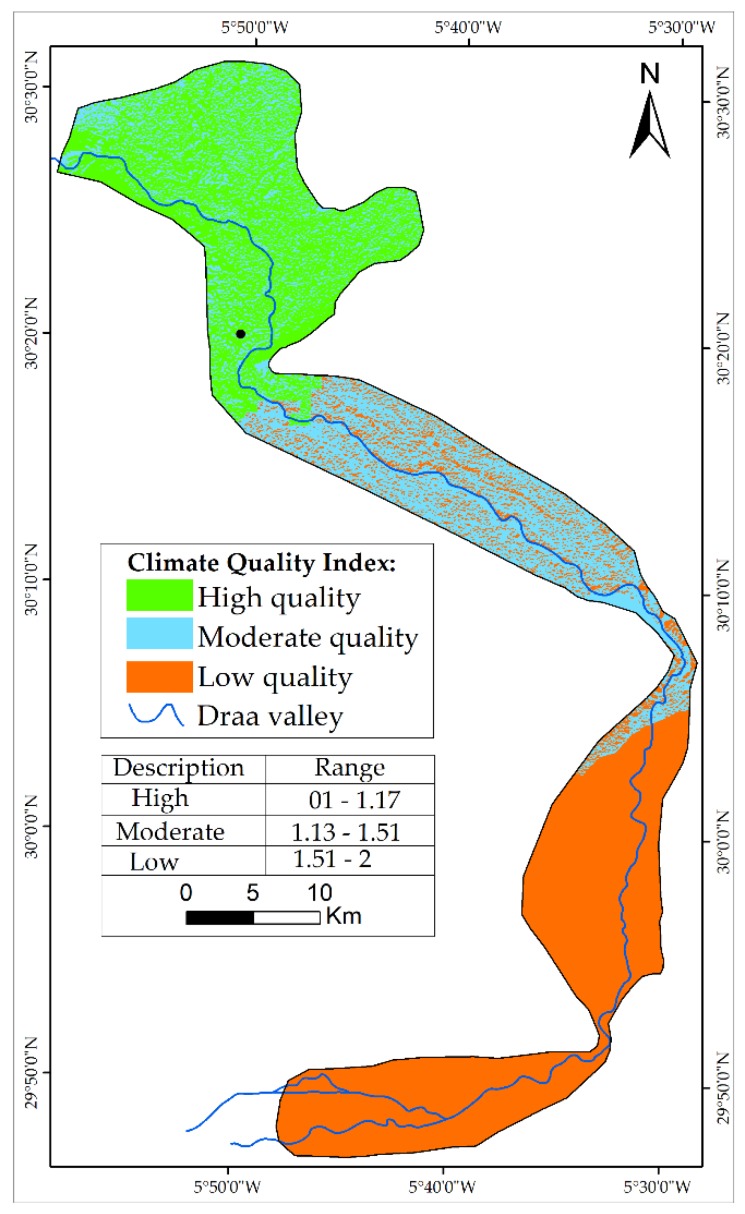
Map of CQI.

An overlay analysis of the three indicators produced the map of CQI (Figure 4). The map illustrates the three main zones with low and high quality of climate. The low quality of climate is presented in the south part of the study area (Mhamid and Ktaoua oases). 

### 3.3. VQI 

VQI was calculated by the combination of the four sub-indicators (Table 3). Plant cover density was calculated from the NDVI and then classified. The NDVI varies from −1 to 1. The values of NDVI were classified into three main classes (less than 0.1; from 0.1 to 0.3; and more than 0.3). The last sub-indicators were derived from the map of land cover. According to field survey, the main land use/cover classes in the study area are pastoral lands, seasonal Saharan vegetation, palm grove, agricultural lands, water, bare land, and buildups. These classes were used as inputs for the supervized classification of S-2 imagery with the maximum likelihood algorithm. The accuracy of the map is 97.3%; approximately 200 sample points were used to validate the accuracy of the map.

**Figure 5:**
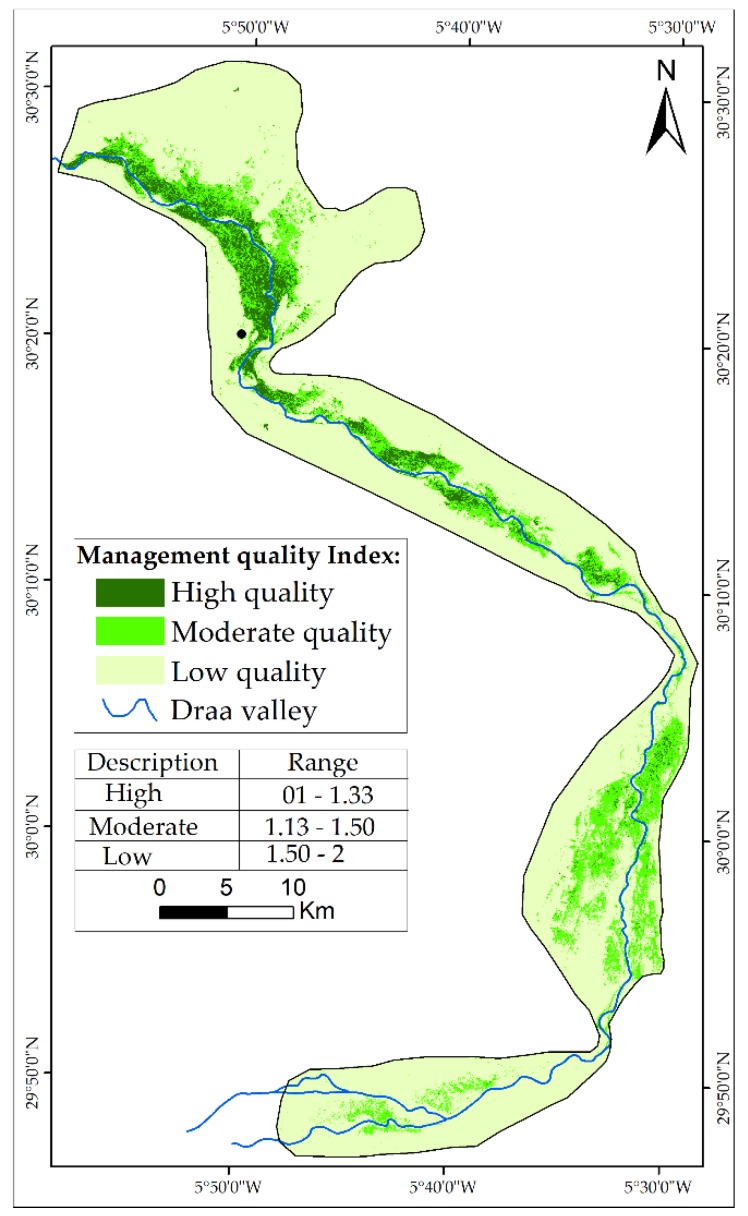
Map of VQI.

The results of the VQI (Figure 5) show that only 5.78% of the study area is classified as high quality and 78.65% is of low quality. High vegetation quality is located generally in the north part of the study area (Ternata oasis). Moreover, a large part of the moderate and low vegetation quality is located in the Ktaoua and Mhamid oases in the southern part of the MDV. 

### 3.4. MQI 

Human pressure (grazing, water supply, and intense agricultural activities) leads to land degradation and desertification. MQI was calculated by the combination of two sub-indicators, namely, human and grazing pressures. For each administrative boundary (named also as rural commune), the number of inhabitants was calculated and then reclassified into three classes (Table 4).

In MDV, two main categories of livestock can be distinguished, namely, domestic (i.e., D’man sheep) and pastoralist livestock (i.e., Rahali goat). Six types of livestock were grouped into classes and were attributed a weight according to their type. The value ‘1’ was assigned for domestic livestock, value ‘2’ for pastoralist livestock and value ‘3’ for camels. The weighted values were attributed according to the influence of grazing on the desertification process. For example, the pastoralist livestock can affect the Saharan grass, a key role for the fixation of dune and wind erosion. 

The two sub-indicators were classified into three classes (Table 5).

**Figure 6:**
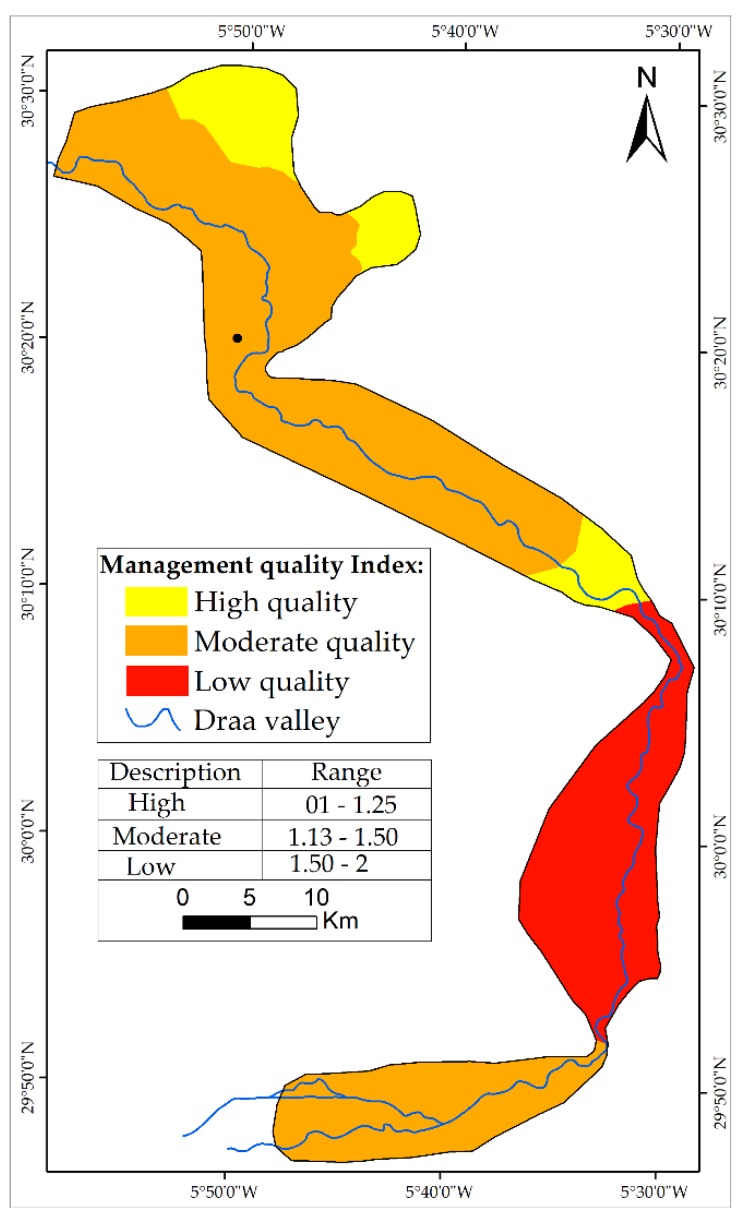
Map of MQI.

The two sub-indicators were classified into three classes of quality, from low to high, and then calculated using geometric mean (Figure 6). The results of the MQI indicate that only 12% of the study area is classified as high quality. In other words, 12% of the study area falls in the zones of low pressure, including human and animal pressures (Table 6). The most affected oases are Ktaoua and Mhamid. 

Table 6 summarizes the areas and portion of each class constituting the four indicators of desertification. The results highlight that vegetation, climate and human activities present low quality in the study area.

### 3.5. ESAI

**Figure 7:**
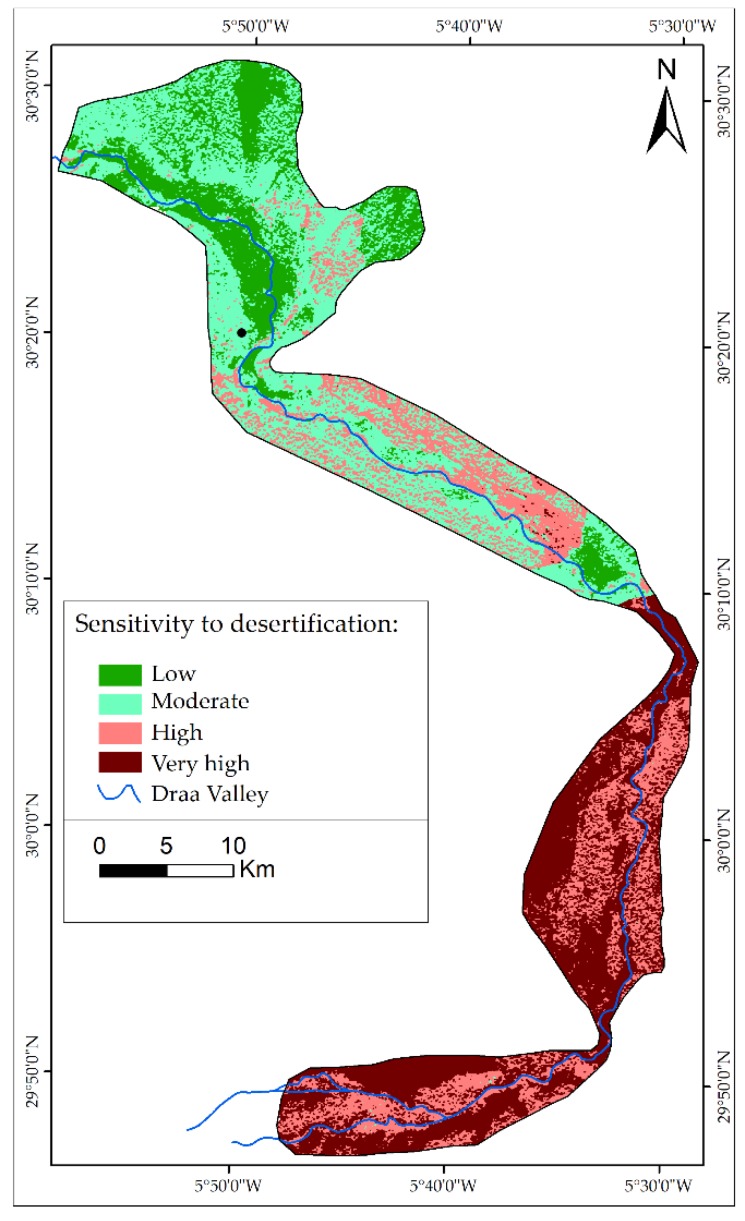
ESAI map.

The sensitivity of the areas to desertification risk was determined by means of four indicators, namely, soil, climate, vegetation and human practices (Figure 7). These combinations were conducted in GIS by using Equation (7). The results obtained revealed that over 50% of the study area is classified as fragile to critical areas (Table 7) and 16.63% are potentially affected.

The final ESAI was calculated by the overlay of the four indicators by integrating the desertification process. The map presents four classes (Figure 7) and shows that the most affected areas are located in the southern part of the study area (Mhamid and Ktaoua oases). According to the map of desertification-sensitive areas, desertification creates a gradient of intensity from north to south. 

**Figure 8:**
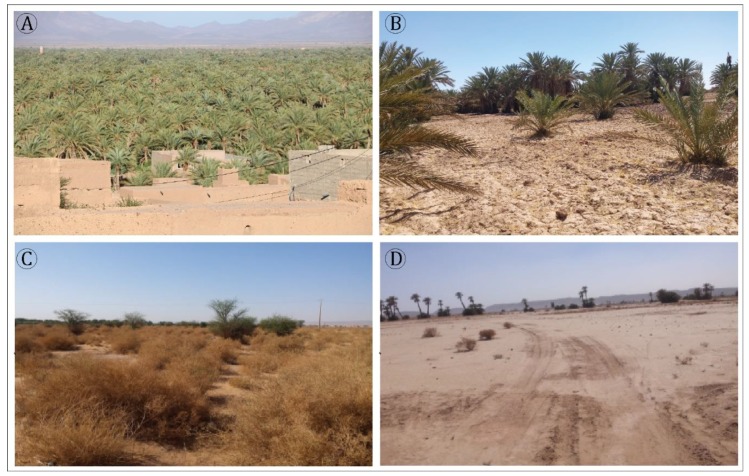
Desertification-sensitive areas: (**A**) low, (**B**) moderate, (**C**) high and (**D**) very high.

The least sensitive areas to desertification are generally located in the Ternata oasis and correspond to the dense palm grove (Figure 8A). This class includes three stages of vegetation (i.e., palm trees, fruit trees and seasonal agricultural vegetation). The moderately sensitive class is located generally in the Fezouata oasis, and this class is characterized by the existence of one level of vegetation, i.e., palm trees (Figure 8B). Moreover, the last two classes, high and very high sensitivity to desertification, are situated in the Ktaoua an M’hamid oases, where the main land cover are sand dunes, bright soils and degraded palm and alfalfa, as shown in Figure 8C and Figure 8D. 

## 4. Discussion

In this research, the MEDALUS approach was adopted because several studies in similar arid regions adopted it; for example, Egypt [6], Iran [50,51], Morocco [21], and Algeria [52]. In this research, we modified and adjusted the original values of the MEDALUS approach proposed by [10]. The brightness parameter, albedo in our case, was added as a factor of calculation of soil quality. The brightness can be an alternative of organic matter data in regions that lack data related to organic matter. 

The aim of any monitoring and assessment of desertification threats is to understand and predict changes and trends over time and space to promote a suitable management system. Furthermore, the MEDALUS approach can be an efficient tool for managing and protecting fragile arid lands, especially oasis areas. The approach is also flexible because it allows users to add, remove, and adjust the sub-indicators as necessary.

The absence of historical data (i.e., climatological and socio-economic data about the soil characteristics) is one of the limitations of ecosystem monitoring and desertification assessment in several developing countries. In the MDV, climatological stations are limited and do not cover the entire study area. The data also contain several gaps and missing values for the time series. Data related to organic matter and soil fertility are not available for the study area. Hence, the freely available database of WorldClim data [28] was used due to the unavailability of a local dataset.

**Figure 9:**
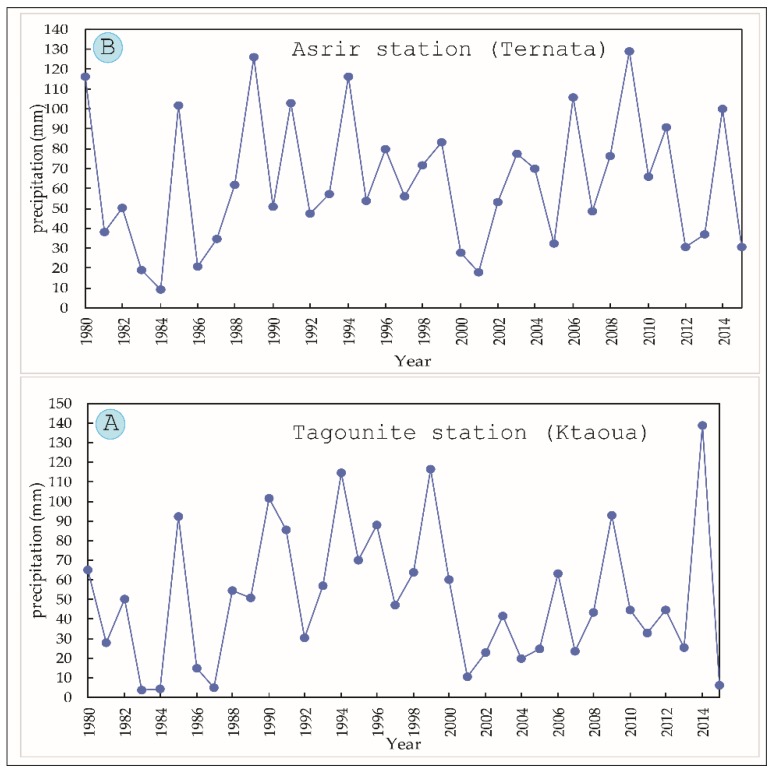
(**A**) Average annual precipitation in Ktaoua; (**B**) average annual precipitation in Ternata between 1980 and 2015.

The MDV is the most degraded area in Morocco [53]. The causes of desertification in this area are due to various factors, including both natural and anthropogenic. The main causes of desertification are climatic factors, characterized by low and variable rainfall (100 m in average). Two patterns of rainfall can be observed in this area, i.e., successions of drought years or exceptional years with floods. Figure 9 shows the average annual precipitation of the two climatological stations of the Ternata and Ktaoua oases. A high intra-annual variability can be seen in the pattern of rainfall for the period of 1980–2015.

**Figure 10:**
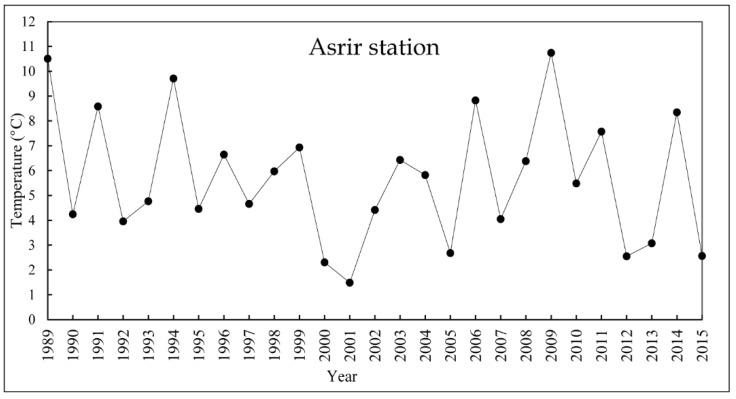
The annual average temperature of the Asrir station (Ternata oasis) 1989–2015.

The pattern of the temperatures is also characterized by the high inter-annual variability. The minimum annual average temperature is 2.55 °C recorded in 2002 against 10.74 °C as the maximum value of the annual average, recorded in 2009 (Figure 10). However, by the analysis of the series of monthly temperatures, a high inter-annual variability was observed. The minimum monthly temperature was recorded in December 2014 (−0.3 °C), the maximal value was also recorded in the same year with a value of 44.2 °C. The high temperatures in the studied area affect the availability of water for the plants caused evaporation and, consequently, the degradation of lands.

Anthropogenic pressure is also the main factor affecting ecosystems, which are already fragile. Human practices are characterized by overgrazing and inadequate agricultural practices. In 2009, the number of camels in Mhamid and Ternata oases was more than 8400. Camel grazing in the pastoral lands degrades the Saharan grassland (a factor of fixation of soils against wind and water erosion). In the last year, the number of wells and motor pumps rapidly increased. The number of pumps was estimated in 1977 and 2011 to be 2000 and more than 10,000, respectively [54], in the MDV. The risk of overexploitation causes water scarcity and increases the salinity in the groundwater system. Water salinity influences the soil quality, thus causing soil salinity.

In 2017, a field study was conducted to collect water samples (90 samples) from different wells around the MDV. Electrical conductivity was used to calculate water salinity. According to our analysis, the average of water salinity was 2.7 g/L in Ternata, 4.36 g/L in Fezouata, 4.36 g/L in Ktaoua, and 5.33 g/L in Mhamid. These results indicate that the concentration of water increases from north to south of the site of study. This finding confirms the result of the map of the sensitive areas. 

Since 2007, the agricultural areas of watermelon farms have increased due to the subvention of the Green Morocco Plan, with which 90% of the fees of agricultural exploitation can be reached [55]. These practices require large quantities of water, thereby causing water scarcity in the Draa catchment and, consequently, the desertification process. 

The first main driving force of desertification in these oases is climatic conditions with high evaporation and low and high variability of rainfall. Furthermore, the socio-economic factors accelerate the degradation of the oases [56]. Due to poverty, the local people cut wood in pastoral lands for domestic purposes or pottery, especially in the Fezouata oasis. The tourism activities are one of the driving forces that lead to the vulnerability of oases [24]. A tourist living at a hotel can consume three times the amount of water used by the local residents [57]. Tourism activities can be an economical alternative that can mobilize the economy of the MDV, provided that the sustainability of natural resources is respected.

Policy strategies and national administrations must focus their subsidies on sustainable agricultural activities that suit arid climates, such as aromatic and medicinal plants. The regeneration of palm date heritage and the adoption of new species with high market value can be an alternative to supplying high quantities of water to certain agricultural types. 

## 5. Conclusions

In this study, ESAs were mapped in the MDV. The MEDALUS approach was adjusted to develop a regional model that could be adapted to the oases located southeast of Morocco. Four composite indices, with each comprising several sub-indicators, were analyzed through a GIS-based approach. Soil, climate, vegetation, and human pressure factors were combined and scored in a GIS to develop a map of desertification-sensitive areas. The results showed that climate factors are the primary sources of desertification and land degradation, combined with high human pressure as the most important indicator for describing desertification sensitivity in the last four oases of the MDV. Climate patterns are characterized by low rainfall and high temperatures, and human activities are characterized by a high consumption of water resources. On the basis of the obtained desertification map, nearly 40% of the study area was classified as potentially sensitive to desertification.

The improvement of the MEDALUS approach in perspective works will be focused on the integration of data related to groundwater quality (groundwater recharge and water salinity). In addition, the integration of soil salinity as a sub-indicator for deriving SQI will be considered. Tourism, as a second principal economic activity in the MDV, will be integrated as an index named tourism pressure. 

Finally, the limitation of desertification research assessment and monitoring in several developing countries (e.g., southeast of Morocco) is the absence of historical data, including climatic (wind direction and speed), pedological (soil salinity, organic matter), and socio-economic data (poverty, gross domestic product). 

## Figures and Tables

**Table 1:** Classes and assigned weighting values for different soil sub-indicators.

Index	Class	Description	Weight
Parent material	Coherent	Limestone granite, quartzite, basalt, conglomerate	1
	Moderately	Unconsolidated scree, granite, rhyolite, gneiss	1.5
	Soft to friable	Clay, marl, sand, superficial formations	2
Slope (%)	<6	Flat to gentle	1
	6–18	Steep	1.5
	18–35	Very gentle	2
Soil depth (cm)	60–100	Deep	1
	31–60	Moderate	1.5
	<30	Shallow	2
Soil Brightness (Albedo)	0–0.2	Somber	1
	0.2–0.25	Moderately bright	1.5
	0.25–1	Bright	2

**Table 2:** Classes and assigned weighting values for different climatic sub-indicators.

Index	Class	Description	Weight
Rainfall (mm)	>85	High	1
	70–85	Moderate	1.5
	55–70	Low	2
Aridity	0.03–1	Low aridity	1
	0.023–0.03	Moderate aridity	1.5
	0.019–0.023	High aridity	2
Aspect	NW–NE	Wet	1
	SW–SE	Dry	2

**Table 3:** VQI sub-indicators and corresponding weight values.

Index	Class	Description	Weight
Fire Risk	Low	Water, bare land	1
	Moderate	Pastoral lands, seasonal Saharan vegetation	1.5
	High	Palm grove, agricultural lands	2
Erosion protection	Low	Palm grove, agricultural lands	1
	Moderate	Pastoral lands, seasonal Saharan vegetation	1.5
	High	bare land, sand dunes	2
Drought resistance	Low	Palm grove, agricultural lands	1
	Moderate	Pastoral lands, seasonal Saharan vegetation	1.5
	High	Bare land, water body	2
Plant cover	Low	>30%	1
	Moderate	10–30%	2
	High	<10%	3

**Table 4:** Demography and livestock census for localities (administrative boundaries) of the study area.

Locality	Demography	Cattle	Sheep D’man	Sheep Rahali	Dairy goat	Rahali goat	Camel
Ktaoua	16,167	80	5284	0	252	12,108	3149
Mhamid	6871	7	4963	2474	1764	5888	5312
Zagora	39,987	102	620	0	490	0	173
Ternata	16,512	152	6656	0	314	0	0
Errouha	10,511	148	3490	0	150	804	4
Fezouata	9416	216	4304	0	0	218	12
Tamgroute	21,574	332	2340	0	72	0	8
Benizoli	18,941	518	2730	0	177	606	0

**Table 5:** MQI sub-indicators and corresponding weights.

Index	Class	Description	Weight
Human pressure (Capeta)	<10,000	Low	1
	10,000–20,000	Moderately dense	1.5
	>20,000	Very dense	2
Grazing pressure	<5500 units	Low	1
	5500–7500 units	Moderately dense	1.5
	>7500 units	Very dense	2

**Table 6:** Areas of class of each sub-indicator.

Indicator	Class	Area (km^2^)	Proportion (%)
SQI	High	369.68	43.21
	Moderate	266.34	31.13
	Low	219.40	25.64
VQI	High	49.58	5.78
	Moderate	133.38	15.55
	Low	674.32	78.65
CQI	High	234.75	27.43
	Moderate	255.06	29.80
	Low	365.93	42.76
MQI	High	108.23	12.62
	Moderate	548.15	63.92
	Low	201.08	23.45

**Table 7:** Results of ESAI.

Index	Description	Surface (km^2^)	Proportion (%)
ESAI	Potentially affected areas	141.85	16.63
	Moderately fragile areas	281.58	33.02
	Highly fragile areas	199.73	23.42
	Highly critical areas	229.56	26.92
